# A child opportunity index in Italy: a pilot proposal

**DOI:** 10.1186/s13052-024-01825-4

**Published:** 2024-12-05

**Authors:** Pietro Ferrara, Domenico Cipolla, Giovanni Corsello, Luca M. Lagalla, Matilde Tantillo, Giusyelisa Galione, Chiara Martorana, Teresa Mazzone, Margherita Zona, Ignazio Cammisa

**Affiliations:** 1 Department of Medicine and Surgery, Università Campus Bio-Medico, Rome, Italy; 2 Operative Research Unit of Pediatrics, Fondazione Policlinico Universitario Campus Bio-Medico, Rome, Italy; 3 Department of Pediatric Emergency, ARNAS Civico, Di Cristina Benfratelli, Palermo, Italy; 4 Department of Sciences for Health Promotion and Mother and Child Care “G.D’Alessandro”, University of Palermo, Palermo, Italy; 5 Pediatrician of Public Health, Rome, Italy; 6 Institute of Pediatric, Catholic University, Rome, Italy

**Keywords:** Child opportunity index, Neighborhoods, Children, Health

## Abstract

**Background:**

The Child Opportunity Index (COI) is a new and innovative tool designed to assess the environment in which children grow up, offering a broad evaluation of the opportunities available to them in different neighborhoods. This initiative aims to ensure improvements in children’s living conditions and future health outcomes.

**Methods:**

The study was performed in the cities of Palermo and Rome. Our Italian COI consists of three main domains: education, health and environment, and economy, each subdivided into specific indicators. We collected information, when available, useful for our indicators from institutional sites and municipal archives. Furthermore, in the city of Rome, we distributed a questionnaire through local pediatricians, collecting data in 2 randomly chosen neighborhoods with questions on children’s health and quality of life, proposing an initial approach that, when implemented using data provided by the government and public and private health institutions, aims to evaluate the correlation between socio-economic opportunities and the psycho-physical health of children, as demonstrated in the literature.

**Results:**

As a result, many aspects, such as the rate of air pollution or the illegal occupation of houses, were not taken into consideration. We therefore consider our COI proposal only a starting model that will have to be implemented once all the necessary information has been obtained. However, what can be deduced from this first descriptive study is how the opportunities in different neighborhoods are not the same for all children. The number of educational opportunities as well as the number of environmental opportunities differs between the various districts and is not homogeneous between different cities or within the same city.

**Conclusions:**

In conclusion, it is not simple to analyze in a scientific manner the child’s health impact of living in different areas. The COI could be a useful and simple tool that can give us this information. Pediatricians could collaborate with institutions to implement intervention plans and to reduce existing differences, social and health inequalities. Future studies will have to implement this pilot study to create and validate an Italian model of COI to be used as a useful tool in children’s assistance.

**Supplementary Information:**

The online version contains supplementary material available at 10.1186/s13052-024-01825-4.

## Background

The Child Opportunity Index (COI) is a new and innovative tool designed to assess the environment in which children grow up, offering a broad evaluation of the opportunities available to them in different neighborhoods [1]. It is structured into three principal domains: education, health and environment, and economic conditions [1, 2]. Regarding the education domain, the COI explores indicators such as the quality of schools and education, access to educational resources and educational attainment rates [1, 2]. It has been demonstrated that these factors are critical in child’s learning outcomes and academic success [3–6]. The health and environment domain of the COI regards the accessibility and quality of healthcare services, the presence of recreational and green spaces, levels of pollution, and neighborhood safety, involving rates of road accidents [7, 8]. These factors have a serious role on physical and mental well-being of children [1]. For example, access to green and sport areas can encourage physical activity, reducing the risk of obesity and metabolic comorbidities, and social interaction [9–12]. The economic domain analyzes employment rates, economic stability, and the availability of economic resources, exploring the material conditions which may support children’s development. It is known that high economic stability in a neighborhood often correlates with better infrastructure and resources that facilitate child development [12, 13].

By integrating these indicators, the COI provides a comprehensive view of the conditions that influence children’s development, identifying neighborhood with very low, low, moderate, high, or very high opportunities and giving a measure of the quality of environments that children experience every day. In this way, the COI has important predictive value as a multidimensional measure of neighborhood living conditions that could guide patient- and community-level interventions, public health planning, and policy to ensure optimal individual health and equal opportunity for all children, regardless of where they live or their race and ethnicity.

Following the American model, the establishment of a COI with the support of the government, institutions, and pediatricians appears increasingly necessary in Italy. This initiative aims to ensure improvements in children’s living conditions and future health outcomes. This purely descriptive pilot study aims to lay the foundation for the creation of an Italian COI in light of the literature and the results from the American experience.

## Materials and methods

Based on the standardized COI in the United States, we developed an Italian pilot model of the COI, incorporating indicators comparable to the American index (Table 1). This model was applied in the cities of Palermo and Rome, with both cities divided into neighborhoods. The city of Palermo has been divided into 8 districts according to the administrative subdivision and into 5 Territorial Assistance Units (PTA). On the other hand, only 9 districts of Rome were included in our study.

**Table 1:** Opportunity indicators in a pilot Italian “Child opportunity Index”

Educational Opportunity	Early childhood and elementary education	• Nursery schools and elementary schools • Middle School diploma
	Secondary and postsecondary education	• High schools • Graduation rate • Years of education
	Educational and social resources	• Municipal libraries • Bookshops (affiliated for the supply of school textbooks) • Cultural and artistic associations • Cinemas and theatres
Health and Environmental Opportunity	Healthy environments	• Access to recreational and sports areas • Access to green spaces • Road accidents
	Health resources	• Health care • General practitioners and paediatricians
Economic Opportunity	Economic opportunities	• Average income

Our Italian COI consists of three main domains: education, health and environment, and economy, each subdivided into specific indicators. The educational domain includes metrics such as school education across various life stages, diploma and degree attainment rates, average years of study, and educational resources. The health and environment domain encompasses access to recreational and green spaces, availability of sports areas, road accident rates, and the quality of healthcare services. The economic domain evaluates the average household income and indirectly assesses the employment rate.

Collectively, these indicators provide a comprehensive overview of the opportunities available to children within a given socio-economic context.

We collected information, when available, useful for our indicators from institutional sites and municipal archives.

Furthermore, in the city of Rome, we distributed a questionnaire through local pediatricians, collecting data in 2 randomly chosen neighborhoods (district 7 and 13) with questions on children’s health and quality of life, proposing an initial approach that, when implemented using data provided by the government and public and private health institutions, aims to evaluate the correlation between socio-economic opportunities and the psycho-physical health of children, as demonstrated in the literature.

Statistical analysis was performed using IBM SPSS Statistics 24.0 software (IBM Corporation, Armonk, NY, USA), through which we conducted a purely descriptive statistical analysis. Percentages and numerical values of the collected data were reported. Additionally, participants’ opinions were documented through direct quotes.

## Results

### District characteristics in Palermo

The geo-demographic characteristics of the eight districts of Palermo (surface area expressed in kilometres^2^ and the resident population, divided into Italian and non-Italian nationalities, updated as of 31/12/2022) are shown in Table 2. The district with the largest extension is the seventh district, while the district with the smallest extension is the first district. The district with the largest population is the eighth district, while the district with the smallest population is the first district. The COI indicators, divided into the 3 domains, which we have analysed if available, are shown in Table 3.

**Table 2:** Geo-demographic characteristics of the eight districts of Palermo

District	Resident Population (Italian nationality)	Resident Population (Other nationality)	Surface area (km^2^)
I	25 516	4283	2,497
II	69 259	1 370	21,39
III	71 580	3 622	20,347
IV	98 443	1 568	26,163
V	108 634	5 237	17,53
VI	70 200	839	23,9
VII	74 689	1 235	32,955
VIII	116 602	5668	15,327

**Table 3:** The analysed COI indicators, divided into the 3 domains

Educational Opportunity- Educational and social resources									
District	Municipal Libraries(*n*°)	Cinemas(*n*°)	Theatres(***n***°)	Schools(*n*°)	Middle school diploma		High school diploma	Graduation	Schooling rate (%)
I	18	1	8	20	7972		5515	4251	42
II	3	1	0	41	25,018		16,785	4742	34,2
III	0	0	1	40	24,338		18,426	6719	38,6
IV	1	1	0	52	31,027		28,687	11,914	44,7
V	3	1	1	67	34,463		30,369	12,800	43,2
VI	2	2	1	34	16,827		23,453	14,307	57,8
VII	2	1	1	43	23,297		21,715	9390	45,4
VIII	7	3	5	39	24,089		35,593	33,281	63,2
**Health and Environmental Opportunity**									
**District**	**Recreational areas (n°)**		**Green spaces (n°)**		**Sport areas (n°)**			**Road accidents (n°)**	
I	13		6		0			215	
II	3		0		2			295	
III	1		0		3			233	
IV	2		2		1			366	
V	1		3		2			388	
VI	0		2		3			354	
VII	1		1		3			264	
VIII	2		6		2			921	
**Economic Opportunity**									
**District**	**Average family income (€)**								
I	41,039								
II	60,960								
III	35,965								
IV	96,512								
V	21,666								
VI	22,736								
VII	126,838								
VIII	136,104								
**Territorial Assistance Unit (PTA)**			**General Practitioners**			**General Pediatricians**			
PTA CENTRO			66			14			
PTA E. ALBANESE			159			28			
PTA BIONDO			147			27			
PTA CASA DEL SOLE			134			20			
PTA GUADAGNA			121			26			

### District characteristics in Rome

The geo-demographic characteristics of the nine districts of Rome (surface area expressed in kilometres^2^ and the resident population, updated as of 31/12/2018) are shown in Table 4. In our analysis, the district with the largest extension is the ninth district, while the district with the smallest extension is the first district. The district with the largest population is the sixth district, while the district with the smallest population is the seventh district. The COI indicators, divided into the 3 domains, which we have analysed, if available, are shown in Table 5.

**Table 4:** Geo-demographic characteristics of the nine districts in Rome (surface area expressed in kilometres^2^ and the resident population, updated as of 31/12/2018)

District	Resident Population	Surface area (km2)
District 1	170,328	20,09
District 4	175,921	48,94
District 5	245,073	26,92
District 6	257,556	113,88
District 7	130,784	45,84
District 9	183,343	183,31
District 12	141,141	73,07
District 13	133,367	66,93
District 14	192,000	133,55

**Table 5:** The analysed COI indicators, divided into the 3 domains

**Educational Opportunity- Educational and social resources**																		
**District**				**Municipal Libraries** (***n***°)				**Bookshops** (***n***°)			**Cultural associations** (***n***°)				**Cinemas** (***n***°)			**Theatres** (***n***°)
District 1				6				8			32				31			62
District 4				3				5			24				6			3
District 5				4				10			5				11			4
District 6				2				12			10				2			1
District 7				4				10			84				7			9
District 9				2				4			68				5			2
District 12				3				8			65				5			7
District 13				2				8			25				2			2
District 14				2				4			9				3			1
**Educational Opportunity- Childhood and secondary Education**																		
**District**	**Nursery and elementary schools (%)**				**High schools (%)**		**Years of education**			**Middle school diploma (%)**			**High school diploma (%)**				**Graduation (%)**	
District 1	50				58		12,5			17,1			32,8				34,1	
District 4	55				21		10,6			26,6			35,3				15	
District 5	61				16		10,3			27			34,6				12,9	
District 6	67				9		9,7			32,2			33,5				8,5	
District 7	75				32		11,2			22,7			37,9				19,4	
District 9	63				20		11,8			20,4			38,5				23,9	
District 12	42				17		11,7			20,6			37				23,9	
District 13	51				13		11			24,3			35,2				19,2	
District 14	48				13		11			24,2			34,6				19,6	
**Health and Environmental Opportunity**																		
**District**			**Recreational areas**			**Green spaces**				**Sport areas**		**General Practitioners**		**General Pediatricians**		**Road accidents**		
District 1			8			210				75		182		18		1.760		
District 4			20			147				64		138		30		552		
District 5			11			130				74		227		34		956		
District 6			8			91				62		129		35		863		
District 7			15			112				50		262		47		967		
District 9			16			210				36		131		27		698		
District 12			5			67				97		111		19		459		
District 13			7			63				59		109		20		655		
District 14			7			55				52		134		23		510		
**Economic Opportunity**																		
**District**		**Average taxable individual income (Italian) €**							**Average taxable individual income (Foreign) €**							**Average family income €**		
District 1		42.497,28							18.429,93							56.989,59		
District 4		22.619,60							12.558,70							34.322,00		
District 5		20.784,26							10.380,04							29.851,92		
District 6		18.571,99							11.113,46							27.778,02		
District 7		25.951,97							13.029,09							38.360,39		
District 9		30.876,94							21.004,36							47.771,96		
District 12		29.415,89							15.059,42							43.187,53		
District 13		25.482,15							13.007,25							37.214,56		
District 14		27.461,98							13.811,06							40.032,77		

### District VII: questionnaire results

The sample in district VII comprised 162 children, of whom 70 (43%) were male with a median age of 10.5 years ± 2.7 years, and 92 (57%) were female with a median age of 10.6 years ± 2.8 years. Of these children, 155 (96%) were of Italian nationality, while 7 (4%) were of other nationalities. Regarding maternal education, 10 mothers had a middle school diploma, 65 had a high school diploma, and 87 had a university degree. For paternal education, 19 fathers had a middle school diploma, 77 had a high school diploma, and 66 had a university degree. Unemployment was reported among 16 (10%) mothers, while 161 fathers (99%) were employed. Additionally, 32% of parents did not consider childcare facilities adequate, 20% were dissatisfied with the education provided to their children in schools, and 39% found the socio-cultural environment in which their children were growing up to be inadequate. Regarding benefits, 73% of the children did not receive school meal or study benefits, and 43% did not benefit from school support or recovery programs. Specific learning disorders were reported in 17 children. Moreover, 12 children did not follow a healthy diet, and 10 did not practice sports. Parental reports also indicated that 14 children did not have access to green areas, 35 to play areas, 100 did not use libraries and bookstores, and 19 did not participate in cultural activities. We also investigated childhood development stages, finding abnormalities in 9 children. Chronic pathologies were present in 13 children, specifically asthma, autism, diabetes type 1 and celiac disease. Additionally, 37 children had at least one visit to the emergency room, and 23 were hospitalized in the last year. In terms of healthcare, 65% of parents considered it inadequate. Regarding mental health, 7 children suffered from behavioral disorders, and 26 children suffered from emotional disorders, specifically anxiety and lack of confidence.

### District XIII: questionnaire results

The sample in district XIII comprised 94 children, of whom 42 (45%) were male with a median age of 9.7 years ± 2.8 years, and 52 (55%) were female with a median age of 9.8 years ± 2.9 years. Of these children, 89 (95%) were of Italian nationality, while 5 (5%) were of other nationalities. Regarding maternal education, 3 mothers had a middle school diploma, 41 had a high school diploma, and 50 had a university degree. For paternal education, 4 fathers had a middle school diploma, 54 had a high school diploma, and 36 had a university degree. Unemployment was reported among 20 (21%) mothers, while all 94 fathers (100%) were employed. Additionally, 33% of parents did not consider childcare facilities adequate, 18% were dissatisfied with the education provided to their children in schools, and 33% found the socio-cultural environment in which their children were growing up to be inadequate. Regarding benefits, 64% of the children did not receive school meal or study benefits, and 41% did not benefit from school support or recovery programs. Specific learning disorders were reported in 7 children. Moreover, 10 children did not follow a healthy diet, and 11 did not practice sports. Parental reports also indicated that 13 children did not have access to green areas, 20 to play areas, 45 did not use libraries and bookstores, and 14 did not participate in cultural activities. We also investigated childhood development stages, finding abnormalities in 4 children. Chronic pathologies were present in 9 children, specifically asthma and celiac disease. Additionally, 29 children had at least one visit to the emergency room, and 17 were hospitalized in the last year. In terms of healthcare, 63% of parents considered it inadequate. Regarding mental health, 3 children suffered from behavioral disorders, and 11 children suffered from emotional disorders, specifically anxiety.

## Discussion

Confronting with a sick child, we pediatricians should consider not only the clinical aspect, but also the several aspects composing child’s daily life which condition the well-being of the children. In this study we evaluated characteristics of the children’s residential area and the available opportunities. As a tool of our analysis, we proposed a pilot model of COI. This descriptive study was based on information obtainable from institutional sites and online municipal archives. As a result, many aspects, such as the rate of air pollution or the illegal occupation of houses, were not taken into consideration. We therefore consider our COI proposal only a starting model that will have to be implemented once all the necessary information has been obtained. In this perspective the role of political and health institutions is therefore fundamental. However, what can be deduced from this first descriptive study is how the opportunities in different neighborhoods are not the same for all children. The number of educational opportunities (schools, libraries, cinemas, etc.) as well as the number of environmental opportunities (access to green areas and sports centers) differs between the various districts and is not homogeneous between different cities or within the same city. Similarly, the different family income could partly reflect the different educational opportunities. For example, considering the city of Rome, the highest family income is recorded in District 1, which also has the highest percentage of university degrees, while the lowest family income is recorded in District 6, which has the lowest levels of educational attainment and years of study. Moreover presumably, as already demonstrated by other studies, race could represent a factor of inequality. It is no coincidence that the family income of non-Italian households is significantly lower than that of Italian households.

The administration of questionnaires provided by pediatricians could represent an additional element to obtain information regarding the quality of life and health of children, as well as to provide parents’ satisfaction ratings towards the services and infrastructures of their neighborhoods. With limited data available, it is not possible to analyze the existence of a potential correlation between neighborhood characteristics and children’s health outcomes. However, this descriptive work aims to lay the foundation for broader-spectrum questionnaires that can investigate the anthropometric characteristics of the population (and the consequent obesity rate, for example), the hospitalization rate, the presence of chronic comorbidities, and their potential correlation with neighborhood opportunities.

Targeting interventions to lower-opportunity neighborhoods and advocating for policies that equitably bolster opportunity may improve child health outcomes, reduce health-related socioeconomic inequities, and decrease health care costs [14–21]. Despite our study being the first aimed at validating an Italian COI, it has several limitations. First, not all characteristics of the American model were analyzed due to the lack of available online data. Additionally, it is necessary to establish cut-off values to categorize neighborhoods into low, medium, and high opportunity areas. The assistance of political institutions would be necessary to obtain homogeneous data. Furthermore for stronger and more accurate results, it should be analyzed not only differences in the same city but differences between whole regions. It would be interesting to compare data from northern and southern part of Italy. Additionally, the small sample size of children in our questionnaires does not allow us to evaluate a potential correlation with the various characteristics of their living environment. Larger samples and more extensive data would be necessary to demonstrate what has already been reported in the literature, namely the positive and negative influences that the surrounding environment can have on children’s health and future outcomes. Finally, a complete and comprehensive evaluation of all COI indicators, some of which were not evaluated due to lack of data in our study, will need to be evaluated in future studies, establishing also the impact of the excluded indicators.

## Conclusion

In conclusion, it is not simple to analyze in a scientific manner the child’s health impact of living in different areas. The COI could be a useful and simple tool that can give us this information. Pediatricians could collaborate with institutions to implement intervention plans and to reduce existing differences, social and health inequalities. Future studies will have to implement this pilot study to create and validate an Italian model of COI to be used as a useful tool in children’s assistance.

## Electronic supplementary material

Below is the link to the electronic supplementary material.

Supplementary Material 1

## Data Availability

All data generated or analyzed during this study are included in this published article.

## Abbreviations

COI: Child Opportunity Index
