# Supplementary material for: A child opportunity index in Italy: a pilot proposal
Source: Ital J Pediatr. 2024 Dec 5;50:258. doi: 10.1186/s13052-024-01825-4 (PMC11622542; doi:10.1186/s13052-024-01825-4)
Supplement: Supplementary file 1 — Supplementary Material 1 [file 13052_2024_1825_MOESM1_ESM.docx]

Dear Editor,

thank you very much for your response and for the kind appreciation of our report.

We have read the Reviewers’ comments and we revised our manuscript in accordance with

their suggestions, hoping we did them in the most proper way.

This is a point-by-point list of replies to each Reviewers’ queries and changes made to our

paper.

Reviewer #1: The authors focus on the Child Opportunity Index (COI), which is a new and innovative tool. The authors detail the composition of the COI and how it is constructed, presenting ideas that are very novel. Certainly，there are also parts that need improvement.

1.In the methodology section, there is no description of any statistical aspects, such as software，the mode of data expression, the source of sample size, and whether it is sufficient to illustrate the issue, which needs further supplementation;

R) We added the missing information.

2.Some indicators have not been included in this study, which needs to be explained as limitation in the summary section;

R) We added the limitation as requested

3.The impact of the excluded indicators on the COI is unknown and further research is needed, which can also be included in the summary.

R) We added the need to also evaluate missing COI data in future studies.
